# Lung X-ray Abnormalities Related to Conventional Gold Mining Activities in Sudan: A Pilot Study

**DOI:** 10.7759/cureus.100426

**Published:** 2025-12-30

**Authors:** Amal Khalil Y Mohammed, Awad Eljeed Abugooda Alobaid, Eldisugi Hassan M Humida, Ahmed Mirghani O Ali, Namarig Alhadi Hamid, Hussain G Ahmed

**Affiliations:** 1 Medicine, Faculty of Medicine, University of Kordofan, El-Obeid, SDN; 2 Medicine, El-Obeid International Hospital, El-Obeid, SDN; 3 Histopathology and Cytology, University of Kordofan, Faculty of Medical Laboratory Sciences (FMLS), El-Obeid, SDN; 4 Medicine, Faculty of Medicine, University of Kordofan, El-Obeid Teaching Hospital, El-Obeid, SDN; 5 Cardiac Catheterization Laboratory, El-Obeid International Hospital, El-Obeid, SDN; 6 Medicine, El-Obeid Teaching Hospital, El-Obeid, SDN; 7 Pathology, Prof Medical Research Consultancy Center, El-Obeid, SDN; 8 Histopathology and Cytology, University of Khartoum, Faculty of Medical Laboratory Sciences (FMLS), Khartoum, SDN

**Keywords:** lung consolidation, lung effusion, lung fibrosis, sudan, traditional gold mining

## Abstract

Background: Traditional gold mining pollutes the world with mercury and silica dust. Silica dust causes respiratory issues, while mercury harms the kidneys and the brain. Mixed exposure can induce these effects and synergistic damage. This study examined lung abnormalities among Sudanese gold workers using radiographic patterns in a clinic-based pilot sample.

Methodology: This study was a cross-sectional, descriptive pilot study conducted in El-Obeid, the capital of North Kordofan State, Sudan. Candidate selection was based solely on their agreement to participate in the study, regardless of demographic characteristics.

Results: A total of 75 men aged 20-62 participated in the study; their mean age was 32.4 years. The most commonly identified lung abnormality was lung fibrosis, followed by consolidation, both fibrosis and consolidation, and effusion, accounting for 44%, 8%, 7%, and 4%, respectively.

Conclusion: The observed radiographic patterns associated with traditional gold mining in Sudan are lung fibrosis, followed by consolidation. The findings primarily reflect individuals with symptoms, which could influence the observed prevalence of abnormalities associated with traditional gold mining practices that pose significant health risks, particularly among the younger population. It is essential to implement formal mining practices that enhance working conditions and provide comprehensive occupational safety training in Sudan.

## Introduction

Occupational diseases, including respiratory disorders, result in significant suffering and economic losses in the workplace. In addition to lung structural disorders, these pollutants impact the immune system and other functional mechanisms within the body. Extended exposure to environmental dust results in the development of severe pulmonary fibrosis [1]. Traditional mining in remote areas results in adverse health outcomes due to both infectious and non-infectious diseases [2]. Exposure to silica elevates the risk of lung infections, including tuberculosis (TB), and can lead to lung damage. As a result, the worker may experience rapidly progressive pneumoconiosis along with progressive massive fibrosis. With disease progression, the individual may encounter dyspnea during physical activity. In later phases, the individual may experience weakness, notable shortness of breath, chest pain, or failure of respiration. Miners face an elevated risk of TB due to factors such as overcrowding, inadequate ventilation in mining camps and sites, substantial exposure to silica dust, co-infection with HIV and TB, and poor nutritional status. Poor ventilation, darkness, and humidity increase TB transmission risk. Miners exhibit a high prevalence of TB, thereby posing significant risks to surrounding communities. The International Labour Organization (ILO) estimates that 2.34 million individuals die annually due to work-related accidents and diseases, including 2.02 million fatalities attributed to work-related diseases [3].

In Sudan, the ongoing armed conflict and its financial consequences have led many youths to leave educational institutions to engage in traditional gold mining, as it represents the most viable source of income. These result in diverse health outcomes that could impact subsequent generations. There is limited literature concerning the effects of mining in Sudan. Available research indicates that conventional gold mining practices in Sudan pose significant health hazards to miners. Community intervention is vital for the protection of individuals in areas dedicated to traditional gold mining. Therefore, this study aimed to evaluate lung abnormalities among Sudanese traditional gold miners using radiographic patterns in a clinic-based pilot sample.

## Materials and methods

Study design and population

The present study is a cross-sectional, descriptive, clinic-based pilot study conducted in El-Obeid, North Kordofan State, Sudan. All participants in the study are individuals currently engaged in traditional gold mining activities. The candidates were chosen based on their willingness to engage in the study, regardless of their demographic characteristics.

Inclusion and exclusion criteria

The candidate must have at least one year of experience as an underground gold miner. A candidate with a known chronic comorbidity before starting work in gold mining was excluded.

Sample size and study measures

All individuals who attended our healthcare clinics with respiratory complaints requiring X-rays as part of the diagnostic process were included.

The study employed a targeted questionnaire to collect patient data, complemented by X-rays to evaluate lung health. A chest X-ray was obtained, and the findings were classified as normal, consolidation, effusion, or both fibrosis and consolidation. X-ray interpretation was conducted by a specialized radiologist along with a consultant chest physician.

Ethics statement

Ethical approval for the study was obtained from the Institutional Research Review Board of the Prof Medical Research Consultancy Center (approval no. HREC 0021/MRCC.1/25). The participants' personal data were kept completely confidential. The data from participants were shared solely in an anonymous format with the research team. This study required that each participant consent to participate before enrollment. The findings aligned with the revised Helsinki Declaration regarding the ethical considerations of research involving human participants.

Statistical analysis

The collected data were initially entered into a data sheet and subsequently imported into IBM SPSS Statistics for Windows, Version 29 (Released 2023; IBM Corp., Armonk, New York). Percentages, frequencies, and tabulations were calculated.

## Results

**Figure 1:**
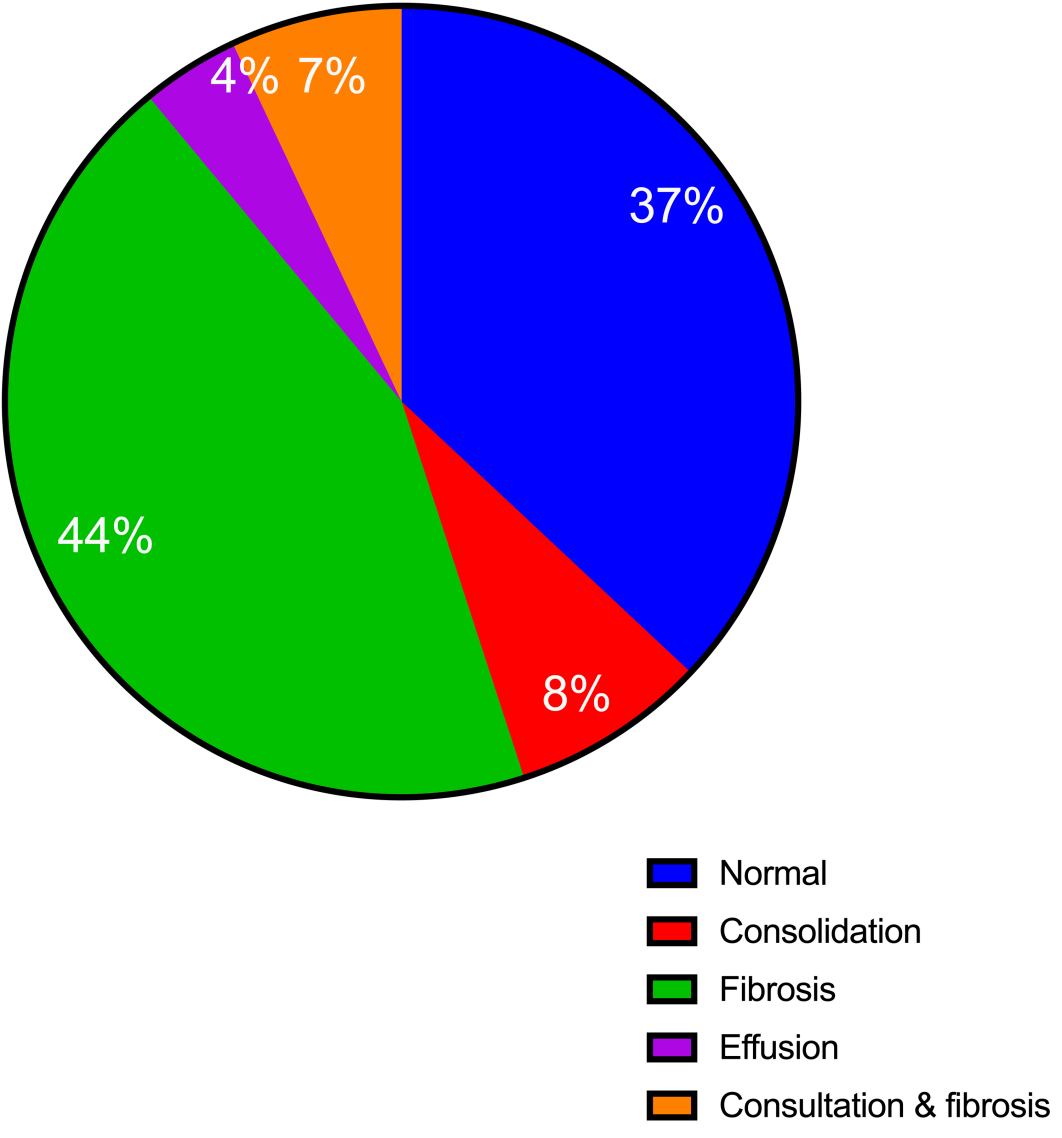
Lung abnormalities in X-rays.

This study included 75 males aged 20-62 years, with a mean age of 32.4 years (SD = 9.9). The most commonly identified lung abnormality on chest X-ray was lung fibrosis, followed by consolidation, both fibrosis and consolidation, and effusion, accounting for 33/75 (44%), 6/75 (8%), 5/75 (7%), and 3/75 (4%), respectively, as shown in Figure 1. More than 80% of the participants are from West Bara province (a rural area).

**Figure 2:**
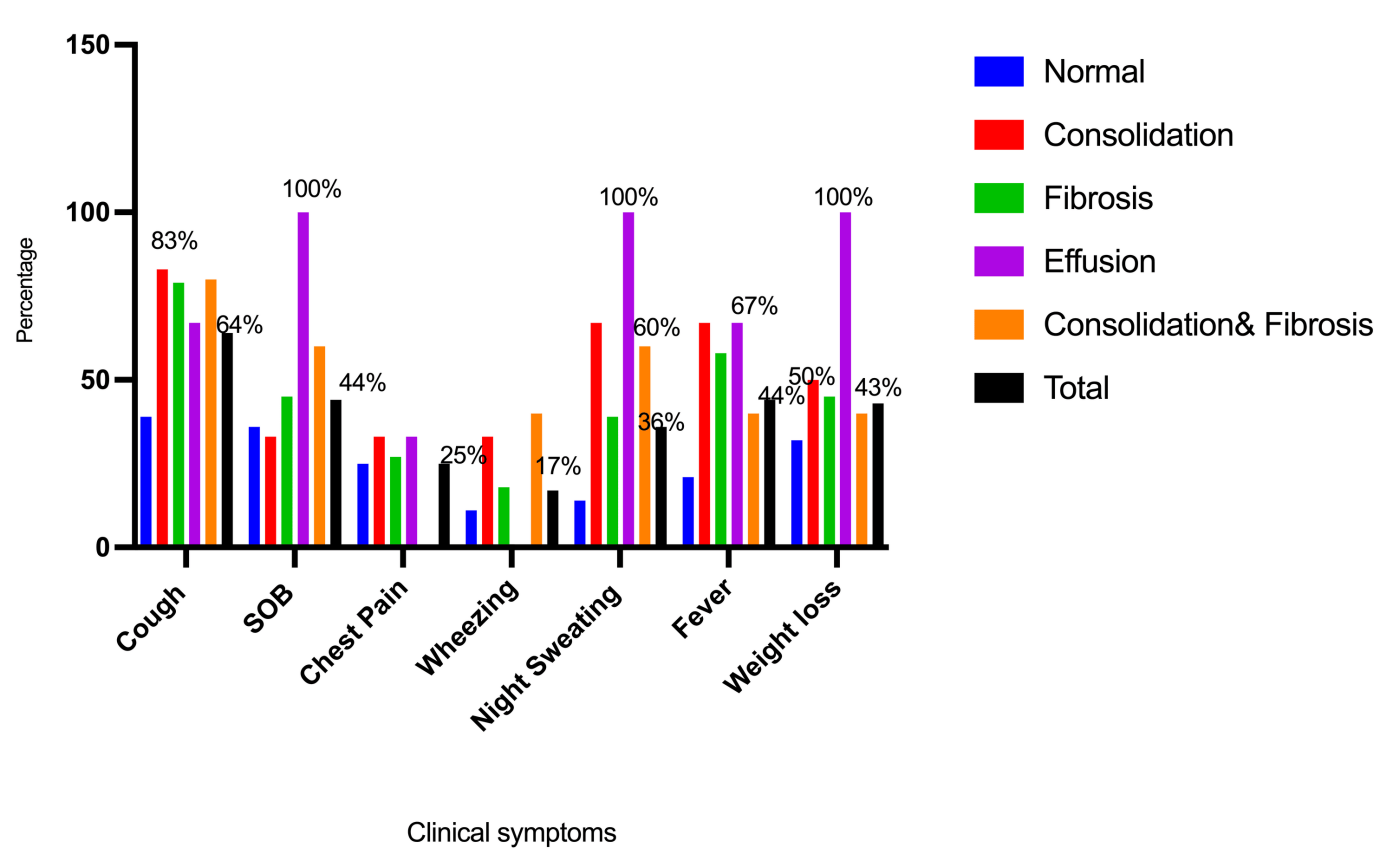
Description of the participants based on their chest X-ray findings and presenting symptoms. SOB: shortness of breath

The most common clinical symptoms associated with this series of patients were cough, followed by shortness of breath (SOB), fever, weight loss, night sweats, chest pain, and wheezing, constituting 48/75 (64%), 33/75 (44%), 33/75 (44%), 32/75 (42.7%), 27/75 (36%), 19/75 (25.3%), and 13/75 (17.3%), respectively. The descriptive observations indicated a strong association between fibrosis and cough, followed by SOB and night sweats, with frequencies of 26 out of 33 (78.8%), 15 out of 33 (45.5%), and 13 out of 33 (39.4%), respectively. Consolidation was more commonly associated with cough (5/6; 83.3%) and night sweats (4/6; 66.7%), as indicated in Table 1 and Figure 2.

**Table 1:** Distribution of the participants by the chest X-ray findings and the presenting symptom.

Variable	Normal n=28	Consolidation n=6	Fibrosis n=33	Effusion n=3	Consolidation & Fibrosis n=5	Total n=75
Cough	11	5	26	2	4	48
Shortness of breath (SOB)	10	2	15	3	3	33
Chest pain	7	2	9	1	0	19
Wheezing	3	2	6	0	2	13
Night sweating	4	4	13	3	3	27
Fever	6	4	19	2	2	33
Weight loss	9	3	15	3	2	32

**Figure 3:**
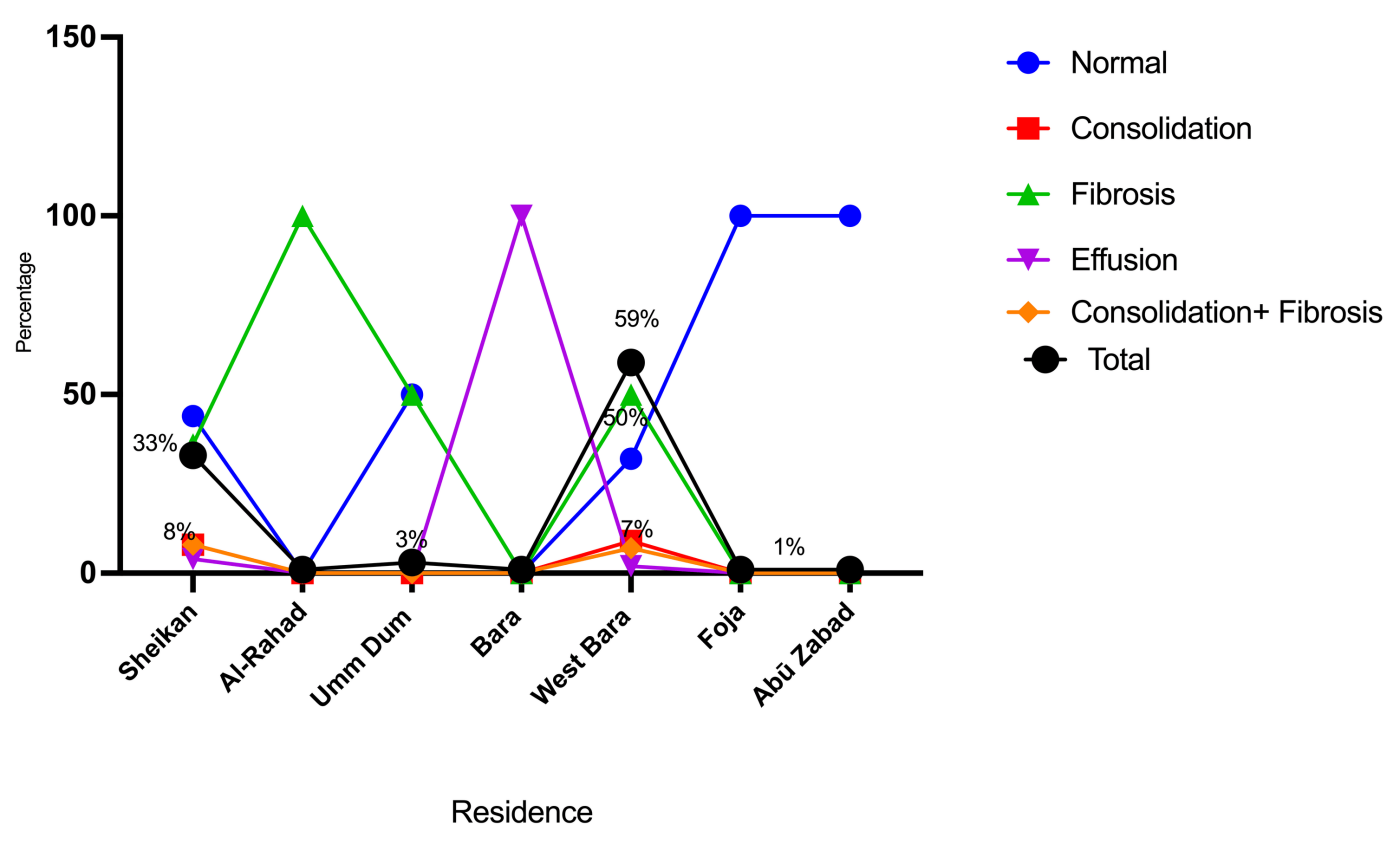
Description of the participants by residence.

**Figure 4:**
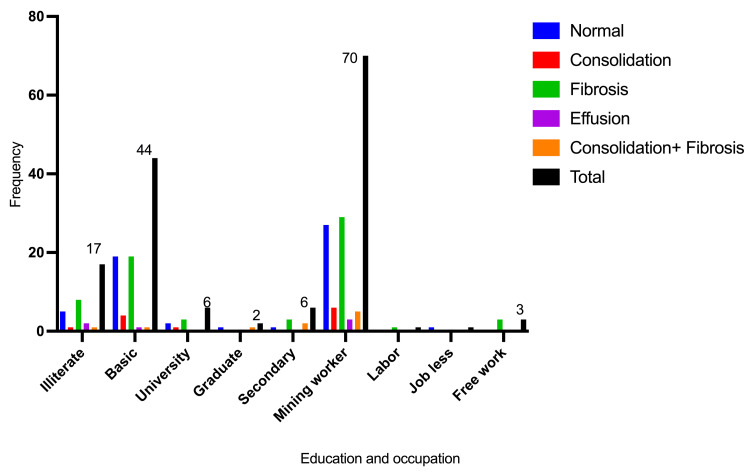
Description of the participants by occupation and education.

Most participants were from rural areas (West Bara province), accounting for 44/75 (58.7%), followed by Sheikan (urban area (El-Obeid city)), 25/75 (33.3%). The majority of patients had basic education, followed by illiterates, representing 44/75 (58.7%) and 17/75 (22.7%), respectively. The great majority of participants were mining workers, 70/75 (93.3%), as indicated in Table 2 and Figures 3-4.

**Table 2:** Distribution of the participants by residence, education, and occupation.

Variable	Normal	Consolidation	Fibrosis	Effusion	Consolidation + Fibrosis	Total
Residence						
Sheikan	11	2	9	1	2	25
Al-Rahad	0	0	1	0	0	1
Umm Dum	1	0	1	0	0	2
Bara	0	0	0	1	0	1
West Bara	14	4	22	1	3	44
Foja	1	0	0	0	0	1
Abū Zabad	1	0	0	0	0	1
Education						
Illiterate	5	1	8	2	1	17
Basic	19	4	19	1	1	44
University	2	1	3	0	0	6
Graduate	1	0	0	0	1	2
Secondary	1	0	3	0	2	6
Occupation						
Mining worker	27	6	29	3	5	70
Labor	0	0	1	0	0	1
Jobless	1	0	0	0	0	1
Free work	0	0	3	0	0	3

## Discussion

The conventional practice of gold mining presents a significant public concern, contributing to a rise in health complications, especially among younger males. The extremely demanding manual labor of stone excavation requires considerable strength, which is why most participants in this task belong to the younger generation, as reflected in the age ranges presented in this study. The results of this study indicated that around 44% of patients developed lung fibrosis, while 7% exhibited both consolidation and fibrosis.

Mine dust refers to all fine solid particles created during mining production. In the mine's actual production environment, dust first appears when mining equipment, such as tunneling machines, crushes or grinds minerals. The loading and unloading of ore materials, along with transportation-related bumps, can damage them, causing dust [4]. It was previously reported that several operating mines had silica levels above 10%, primarily from limestone and rock stratum dust. Underground coal miners can contract black lung and silicosis from coal mine dust. The extent of repairable coal mine dust, along with its intensity and ingredients, directly affects lung cellular damage and inflammation [5].

The predominant occupational ailment among coal miners is pneumoconiosis, resulting from prolonged inhalation and accumulation of coal dust. The initial phase is inconspicuous, whereas pulmonary fibrosis may develop during the intermediate and advanced stages, exacerbating the patient's discomfort levels and risk. A multitude of factors contribute to the complex etiology of pneumoconiosis. While coal dust is believed to contribute to various forms of pneumoconiosis, the influence of its content, particle size, morphology, and concentration on its pathophysiology remains inadequately investigated. Timely diagnosis is essential for patients with pneumoconiosis because the disease is irreversible. No established criteria exist for the early prediction of pneumoconiosis in coal workers [6]. Coal workers' pneumoconiosis is an occupational disease that has a substantial negative impact on miners' health and safety. Nonetheless, the molecular and cellular mechanisms underlying the alterations associated with lung inflammation and fibrosis induced by coal dust are poorly understood [7]. Among mine workers, DNA methylation and oxidative damage were associated with blood levels of titanium, phosphorus, salt, aluminum, iron, sulfur, copper, chromium, zinc, chlorine, calcium, and potassium. Genetic instability, global DNA hypermethylation, and environmental exposure in coal-mining areas interact, necessitating comprehensive mitigation [8].

In sub-Saharan Africa, miners exhibit a greater prevalence of TB (3000-7000 cases per 100,000 miners annually) than any other occupational group globally. Despite being preventable, silicosis remains a prevalent global affliction that impacts mining workers, contributing to illness and mortality [9]. The prevalence of TB among gold miners in South Africa was three times higher than that of the general population. The global TB study indicated that 10.6 million individuals were anticipated to have TB in 2022, reflecting a 4.5% rise from 2021, with 1.1 million fatalities attributed to TB, including 187,000 among HIV-positive individuals [10]. Some countries have reported a prevalence of TB among miners that is 8-10 times higher than in the rest of the community. The spread of TB escalates in inadequately ventilated, dim, and humid environments, posing a significant risk to adjacent communities [11]. A study examined respiratory problems among gold miners in Ghana and identified a strong correlation between age, educational attainment, marital status, alcohol consumption, and respiratory disorders [12].

The findings of this study indicate several clinical manifestations associated with this group of gold mining workers, including chronic cough, chest pain, wheezing, fever with night sweats, and weight loss. Individuals engaged in mining often present with symptoms including a chronic cough (with or without sputum), wheezing, difficulty in breathing (dyspnea), and a sensation of constriction in the chest [13]. In a study of mine industry workers, the most common symptoms reported were fatigue, headache, sweating, and dark-colored urine, with 77% of respondents indicating the presence of at least one symptom [14]. In the current study, lung fibrosis was reported in 47% of the illiterate persons investigated. A study among former and current miners in South Africa also found a high prevalence of respiratory symptoms, particularly among former miners. Pneumoconiosis in the United States has also been associated with a high prevalence of respiratory disorders and symptoms among miners: 28% had cough, 32% had phlegm, 21% had chronic bronchitis, 22% had breathlessness, and 27% had wheezing [15]. It was also reported that a sub-Saharan African nation revealed a substantial correlation between age, educational attainment, marital status, alcohol consumption, and respiratory disorders [16]. In the current study, lung fibrosis showed a high prevalence of respiratory symptoms among miners, with a 54.1% prevalence of cough, 46% SOB, 47% chest pain, 46% wheezing, 48% night sweating, 58% fever, and 47% weight loss.

The leading preventable cause of morbidity and mortality globally is cigarette smoking. The impact of cigarette smoking on gold miners' health makes it more difficult, with an increase in respiratory symptoms, tissue changes associated with emphysema/chronic bronchitis, and greater airflow limitations on spirometry [17]. In the current study, the symptoms of the lungs might be related to tobacco smoking and a longer duration of consumption, which may act as a confounding factor in this pilot assessment. Smoking is linked to chronic inflammation and lung damage, which can lead to pulmonary fibrosis. It should be regarded as a confounding factor when examining lung fibrosis related to environmental or workplace pollutants. This limitation will be taken into account in future evaluations. Cigarette smoking has been shown to interact with occupational exposure to a variety of particulates [18]. The habit of cigarette smoking has been shown to significantly affect the health of miners, notably altering radiologic and histological indicators closely linked to interstitial lung diseases and emphysema [19]. Individuals exposed to silica dust significantly increase their mortality risk due to cigarette smoking. Reducing mortality risk among individuals exposed to silica may necessitate smoking cessation and the regulation of silica dust concentrations [20]. While this study provides previously unavailable data on traditional gold mining in Sudan, its limitations include its status as a pilot study, the lack of a control group, and the assessment of mining dust pollutants.

## Conclusions

Traditional gold mining has a significant impact on the health of Sudan's younger generation. The findings from this pilot, clinic-based sample suggest that lung fibrosis is prevalent among this segment of the population. The risk of developing lung fibrosis increases with prolonged exposure to mining dust.
